# 2′-Carb­oxy­meth­oxy-4,4′-bis­(3-methyl­but-2-en­yloxy)chalcone

**DOI:** 10.1107/S1600536811008300

**Published:** 2011-03-09

**Authors:** Yan-Shu Liang, Shuai Mu, Jing-Yang Wang, Deng-Ke Liu

**Affiliations:** a Tianjin University of Commerce, Tianjin 300134, People’s Republic of China; b School of Chemical Engineering and Technology, Tianjin University, Tianjin 300072, People’s Republic of China; c Tianjin Institute of Pharmaceutical Research, Tianjin 300193, People’s Republic of China

## Abstract

In the title compound, C_27_H_30_O_6_, also known as sofalcone, an anti-ulcer agent used for the protection of gastric mucosa­, the two benzene rings form a dihedral angle of 6.78 (11)°. Inter­molecular O—H⋯O hydrogen bonds link the mol­ecules into centrosymmetric dimers, which are further linked by weak C—H⋯O inter­actions into ribbons propagated in [2
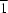
0]. Finally, π–π inter­actions between the benzene rings [centroid–centroid distance = 3.583 (13) Å] stabilize the crystal packing.

## Related literature

For background to the bioactivity and applications of the title compound, see: Tanaka *et al.* (2009 ▶). For a related structure, see: Cheng *et al.* (2007 ▶). For the preparation of the title compound, see: Kyogoku *et al.* (1978 ▶, 1979 ▶); Liu *et al.* (2009 ▶). For standard bond lengths, see: Allen *et al.* (1987 ▶).
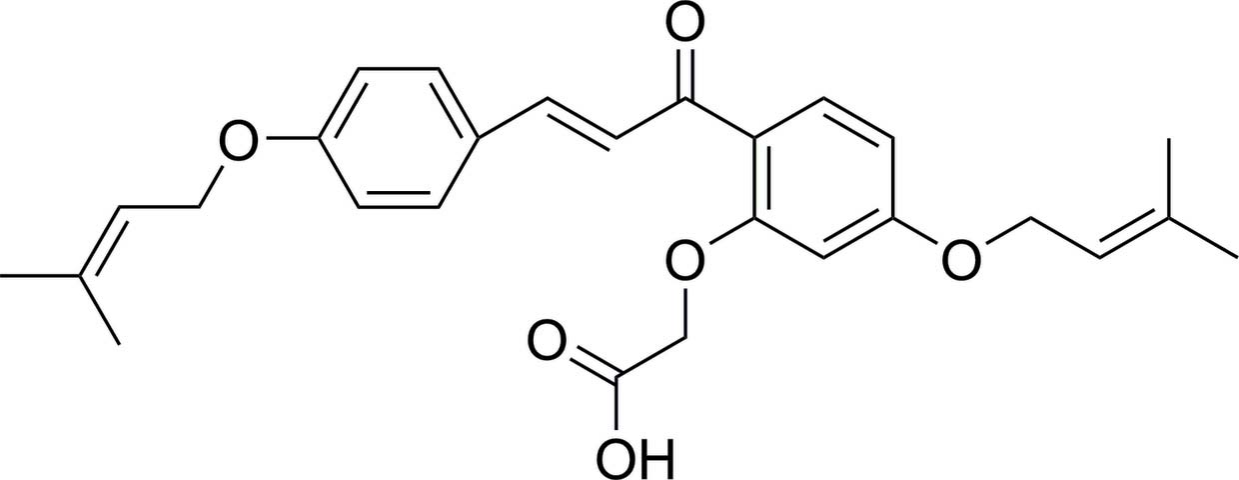


## Experimental

### 

#### Crystal data

C_27_H_30_O_6_
*M* *_r_* = 450.51
Triclinic, 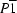

*a* = 7.4496 (15) Å
*b* = 12.195 (2) Å
*c* = 13.085 (3) Å
α = 88.39 (3)°
β = 78.53 (3)°
γ = 86.99 (3)°
*V* = 1163.3 (4) Å^3^
*Z* = 2
Mo *K*α radiation
μ = 0.09 mm^−1^
*T* = 113 K
0.12 × 0.10 × 0.08 mm

#### Data collection

Rigaku Saturn diffractometer
Absorption correction: multi-scan (*CrystalClear*; Rigaku/MSC, 2005 ▶) *T*_min_ = 0.989, *T*_max_ = 0.993
11767 measured reflections
4097 independent reflections
2371 reflections with *I* > 2σ(*I*)
*R*_int_ = 0.078

#### Refinement

*R*[*F* ^2^ > 2σ(*F* ^2^)] = 0.048
*wR*(*F* ^2^) = 0.116
*S* = 0.92
4097 reflections
303 parameters
H-atom parameters constrained
Δρ_max_ = 0.22 e Å^−3^
Δρ_min_ = −0.22 e Å^−3^

### 

Data collection: *CrystalClear* (Rigaku/MSC, 2005 ▶); cell refinement: *CrystalClear*; data reduction: *CrystalClear*; program(s) used to solve structure: *SHELXS97* (Sheldrick, 2008 ▶); program(s) used to refine structure: *SHELXL97* (Sheldrick, 2008 ▶); molecular graphics: *SHELXTL* (Sheldrick, 2008 ▶); software used to prepare material for publication: *SHELXTL*.

## Supplementary Material

Crystal structure: contains datablocks global, I. DOI: 10.1107/S1600536811008300/cv5057sup1.cif

Structure factors: contains datablocks I. DOI: 10.1107/S1600536811008300/cv5057Isup2.hkl

Additional supplementary materials:  crystallographic information; 3D view; checkCIF report

## Figures and Tables

**Table 1:** Hydrogen-bond geometry (Å, °)

*D*—H⋯*A*	*D*—H	H⋯*A*	*D*⋯*A*	*D*—H⋯*A*
O4—H4⋯O3^i^	0.82	1.83	2.6474 (19)	176
C3—H3⋯O5^ii^	0.93	2.56	3.456 (2)	162

Symmetry codes: (i) 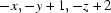; (ii) 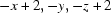.

**Fig. 1.**
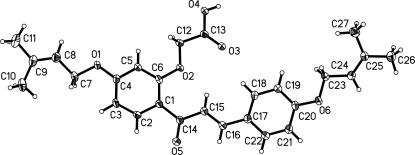
The molecular structure of (I), with the atom-numbering scheme and 50% probability displacement ellipsoids.

## supplementary crystallographic information

### Comment

The title compound, (I), is a chalcone derivative also named sofalcone. It is an anti-ulcer agent used for the protection of gastric mucosa, although its precise molecular mechanism has not been completely understood (Tanaka *et al.*, 2009). The crystal structrue of 1-[2-Hydroxy-4-(3-methylbut-2-enyloxy)-phenyl]ethan-1-one, an intermediate in the synthesis of sofalcone, has been reported by Cheng *et al.* (2007). Now, we present the crystal structure of the title compound.

In (I) (Fig. 1), all bond lengths and angles are within normal ranges (Allen *et al.*, 1987). In the molecule, two benzene rings form a dihedral angle of 6.78 (11)°. Due to the *p*-π conjugation, the C*_sp_*^2^—O bonds [O1—C4 = 1.355 (2) Å and O6—C20 = 1.362 (2) Å] are significantly shorter than the C*_sp_*^3^—O bonds [O1—C7 = 1.441 (2) Å and O6—C23 = 1.442 (2) Å]. Intermolecular O—H···O hydrogen bonds link the molecules into centrosymmetric dimers, which are further linked by the weak C—H···O interactions into ribbons propagated in direction [2–10] (Table 1). Finally, π-π interactions between the benzene rings [centroid-to-centroid distance of 3.583 (13) Å] stabilize the crystal packing.

### Experimental

Several methods have been reported to prepare the title compound (Kyogoku *et al.*, 1978; Kyogoku *et al.*, 1979; Liu *et al.*, 2009). Our experiment was carried out according to the method of Kyogoku *et al.* (1978) to get crude product as yellow powder. The powder (10 g) and activated carbon (1 g) were then dissolved in acetonitrile (80 ml) and the mixture was heated to reflux for 10 min. After filtration, the mixture was standing under room temperature for 24 h, then yellow crystals were generated slowly.

### Refinement

All H atoms were positioned geometrically and refined using a riding model, with d(C—H) = 0.93 - 0.97 Å and d(O—H) = 0.82 Å, and *U*_iso_(H) = 1.2-1.5 *U*_eq_ of the parent atom.

### Crystal data

**Table:**

C_27_H_30_O_6_	*Z* = 2
*M**_r_* = 450.51	*F*(000) = 480
Triclinic, *P*1	*D*_x_ = 1.286 Mg m^−^^3^
Hall symbol: -P 1	Mo *K*α radiation, λ = 0.71073 Å
*a* = 7.4496 (15) Å	Cell parameters from 3474 reflections
*b* = 12.195 (2) Å	θ = 2.3–27.9°
*c* = 13.085 (3) Å	µ = 0.09 mm^−^^1^
α = 88.39 (3)°	*T* = 113 K
β = 78.53 (3)°	Prism, yellow
γ = 86.99 (3)°	0.12 × 0.10 × 0.08 mm
*V* = 1163.3 (4) Å^3^	

### Data collection

**Table:**

Rigaku Saturn diffractometer	4097 independent reflections
Radiation source: rotating anode	2371 reflections with *I* > 2σ(*I*)
confocal	*R*_int_ = 0.078
ω scans	θ_max_ = 25.0°, θ_min_ = 1.7°
Absorption correction: multi-scan (*CrystalClear*; Rigaku/MSC, 2005)	*h* = −8→8
*T*_min_ = 0.989, *T*_max_ = 0.993	*k* = −14→14
11767 measured reflections	*l* = −15→15

### Refinement

**Table:**

Refinement on *F*^2^	Primary atom site location: structure-invariant direct methods
Least-squares matrix: full	Secondary atom site location: difference Fourier map
*R*[*F*^2^ > 2σ(*F*^2^)] = 0.048	Hydrogen site location: inferred from neighbouring sites
*wR*(*F*^2^) = 0.116	H-atom parameters constrained
*S* = 0.92	*w* = 1/[σ^2^(*F*_o_^2^) + (0.0421*P*)^2^] where *P* = (*F*_o_^2^ + 2*F*_c_^2^)/3
4097 reflections	(Δ/σ)_max_ < 0.001
303 parameters	Δρ_max_ = 0.22 e Å^−^^3^
0 restraints	Δρ_min_ = −0.22 e Å^−^^3^

### Special details

**Table:**

Geometry. All e.s.d.'s (except the e.s.d. in the dihedral angle between two l.s. planes) are estimated using the full covariance matrix. The cell e.s.d.'s are taken into account individually in the estimation of e.s.d.'s in distances, angles and torsion angles; correlations between e.s.d.'s in cell parameters are only used when they are defined by crystal symmetry. An approximate (isotropic) treatment of cell e.s.d.'s is used for estimating e.s.d.'s involving l.s. planes.
Refinement. Refinement of *F*^2^ against ALL reflections. The weighted *R*-factor *wR* and goodness of fit *S* are based on *F*^2^, conventional *R*-factors *R* are based on *F*, with *F* set to zero for negative *F*^2^. The threshold expression of *F*^2^ > σ(*F*^2^) is used only for calculating *R*-factors(gt) *etc*. and is not relevant to the choice of reflections for refinement. *R*-factors based on *F*^2^ are statistically about twice as large as those based on *F*, and *R*- factors based on ALL data will be even larger.

### Fractional atomic coordinates and isotropic or equivalent isotropic displacement parameters (Å^2^)

**Table:**

	*x*	*y*	*z*	*U*_iso_*/*U*_eq_	
O1	0.45375 (16)	−0.07058 (11)	1.21292 (10)	0.0295 (4)	
O2	0.39551 (15)	0.25262 (10)	1.01329 (10)	0.0245 (3)	
O3	0.19896 (16)	0.42849 (11)	0.96442 (10)	0.0275 (4)	
O4	−0.04272 (16)	0.38360 (11)	1.08729 (10)	0.0281 (4)	
H4	−0.0869	0.4422	1.0692	0.042*	
O5	0.96148 (17)	0.22153 (11)	0.88715 (11)	0.0325 (4)	
O6	0.52436 (16)	0.79502 (10)	0.64049 (10)	0.0290 (4)	
C1	0.6943 (2)	0.16654 (15)	1.00089 (14)	0.0206 (4)	
C2	0.7978 (2)	0.07896 (15)	1.03331 (15)	0.0232 (5)	
H2	0.9231	0.0750	1.0059	0.028*	
C3	0.7285 (2)	−0.00236 (16)	1.10304 (14)	0.0239 (5)	
H3	0.8045	−0.0591	1.1219	0.029*	
C4	0.5426 (2)	0.00300 (15)	1.14398 (14)	0.0225 (5)	
C5	0.4324 (2)	0.08869 (15)	1.11472 (14)	0.0228 (5)	
H5	0.3075	0.0921	1.1429	0.027*	
C6	0.5055 (2)	0.16926 (15)	1.04415 (14)	0.0214 (5)	
C7	0.5612 (3)	−0.16271 (16)	1.24391 (16)	0.0302 (5)	
H7A	0.6645	−0.1375	1.2701	0.036*	
H7B	0.6075	−0.2098	1.1850	0.036*	
C8	0.4368 (3)	−0.22398 (16)	1.32763 (15)	0.0285 (5)	
H8	0.3166	−0.1963	1.3476	0.034*	
C9	0.4851 (3)	−0.31441 (16)	1.37529 (15)	0.0291 (5)	
C10	0.6741 (3)	−0.36753 (17)	1.35051 (17)	0.0428 (6)	
H10A	0.7473	−0.3280	1.2941	0.064*	
H10B	0.6672	−0.4421	1.3306	0.064*	
H10C	0.7290	−0.3665	1.4109	0.064*	
C11	0.3521 (3)	−0.37149 (18)	1.45850 (16)	0.0434 (6)	
H11A	0.2355	−0.3316	1.4694	0.065*	
H11B	0.3977	−0.3747	1.5222	0.065*	
H11C	0.3381	−0.4447	1.4371	0.065*	
C12	0.2107 (2)	0.26291 (15)	1.06756 (15)	0.0234 (5)	
H12A	0.2065	0.2641	1.1421	0.028*	
H12B	0.1442	0.2008	1.0530	0.028*	
C13	0.1247 (2)	0.36717 (15)	1.03284 (15)	0.0223 (5)	
C14	0.7976 (2)	0.24378 (15)	0.92286 (15)	0.0222 (5)	
C15	0.7105 (3)	0.34591 (15)	0.88957 (15)	0.0250 (5)	
H15	0.5896	0.3648	0.9202	0.030*	
C16	0.8002 (3)	0.41176 (15)	0.81682 (15)	0.0246 (5)	
H16	0.9221	0.3914	0.7899	0.030*	
C17	0.7269 (3)	0.51316 (15)	0.77462 (15)	0.0238 (5)	
C18	0.5463 (2)	0.55068 (15)	0.80792 (15)	0.0265 (5)	
H18	0.4715	0.5119	0.8605	0.032*	
C19	0.4741 (3)	0.64361 (16)	0.76561 (15)	0.0263 (5)	
H19	0.3522	0.6665	0.7893	0.032*	
C20	0.5842 (3)	0.70289 (15)	0.68747 (15)	0.0237 (5)	
C21	0.7656 (3)	0.66794 (16)	0.65364 (15)	0.0267 (5)	
H21	0.8406	0.7075	0.6017	0.032*	
C22	0.8355 (3)	0.57460 (16)	0.69672 (15)	0.0274 (5)	
H22	0.9577	0.5521	0.6733	0.033*	
C23	0.3328 (2)	0.82802 (16)	0.67028 (15)	0.0291 (5)	
H23A	0.3008	0.8407	0.7447	0.035*	
H23B	0.2576	0.7710	0.6537	0.035*	
C24	0.3013 (3)	0.93145 (17)	0.61086 (16)	0.0361 (6)	
H24	0.3969	0.9533	0.5583	0.043*	
C25	0.1492 (3)	0.99420 (16)	0.62685 (15)	0.0293 (5)	
C26	0.1281 (3)	1.09886 (17)	0.56591 (17)	0.0453 (7)	
H26A	0.2386	1.1096	0.5155	0.068*	
H26B	0.1048	1.1596	0.6126	0.068*	
H26C	0.0273	1.0942	0.5308	0.068*	
C27	−0.0158 (3)	0.96821 (18)	0.70712 (16)	0.0382 (6)	
H27A	0.0093	0.9020	0.7442	0.057*	
H27B	−0.1173	0.9584	0.6737	0.057*	
H27C	−0.0457	1.0276	0.7552	0.057*	

### Atomic displacement parameters (Å^2^)

**Table:**

	*U*^11^	*U*^22^	*U*^33^	*U*^12^	*U*^13^	*U*^23^
O1	0.0232 (7)	0.0239 (8)	0.0385 (9)	0.0037 (6)	−0.0028 (6)	0.0157 (7)
O2	0.0173 (7)	0.0230 (8)	0.0315 (8)	0.0048 (6)	−0.0031 (6)	0.0071 (6)
O3	0.0187 (7)	0.0253 (8)	0.0359 (8)	0.0027 (6)	−0.0011 (6)	0.0096 (7)
O4	0.0198 (8)	0.0236 (9)	0.0375 (9)	0.0061 (6)	−0.0010 (6)	0.0096 (7)
O5	0.0204 (8)	0.0287 (9)	0.0441 (9)	0.0042 (7)	0.0007 (7)	0.0110 (7)
O6	0.0252 (8)	0.0252 (8)	0.0331 (8)	0.0073 (6)	−0.0015 (6)	0.0106 (6)
C1	0.0188 (10)	0.0176 (11)	0.0252 (11)	0.0017 (8)	−0.0048 (8)	0.0007 (9)
C2	0.0160 (10)	0.0222 (12)	0.0310 (12)	0.0007 (9)	−0.0045 (9)	0.0023 (9)
C3	0.0254 (11)	0.0167 (11)	0.0299 (12)	0.0042 (9)	−0.0086 (9)	0.0053 (9)
C4	0.0251 (11)	0.0180 (11)	0.0242 (11)	−0.0012 (9)	−0.0051 (9)	0.0046 (9)
C5	0.0186 (10)	0.0224 (12)	0.0267 (11)	0.0023 (9)	−0.0045 (8)	0.0034 (9)
C6	0.0233 (11)	0.0168 (11)	0.0251 (11)	0.0044 (9)	−0.0089 (9)	0.0011 (9)
C7	0.0318 (12)	0.0213 (12)	0.0367 (13)	0.0062 (9)	−0.0081 (10)	0.0077 (10)
C8	0.0327 (12)	0.0244 (13)	0.0274 (12)	−0.0007 (10)	−0.0041 (9)	0.0008 (10)
C9	0.0424 (13)	0.0218 (12)	0.0242 (11)	−0.0040 (10)	−0.0089 (10)	0.0018 (9)
C10	0.0554 (15)	0.0256 (14)	0.0482 (15)	0.0045 (11)	−0.0152 (12)	0.0100 (11)
C11	0.0607 (16)	0.0346 (15)	0.0361 (13)	−0.0087 (12)	−0.0123 (12)	0.0117 (11)
C12	0.0157 (10)	0.0236 (12)	0.0295 (11)	0.0020 (9)	−0.0024 (8)	0.0024 (9)
C13	0.0166 (11)	0.0220 (12)	0.0285 (11)	0.0006 (9)	−0.0055 (9)	0.0012 (9)
C14	0.0221 (11)	0.0182 (11)	0.0264 (11)	0.0005 (9)	−0.0059 (9)	0.0014 (9)
C15	0.0215 (11)	0.0206 (12)	0.0319 (12)	0.0015 (9)	−0.0042 (9)	0.0044 (9)
C16	0.0216 (11)	0.0211 (12)	0.0300 (12)	0.0025 (9)	−0.0036 (9)	0.0023 (9)
C17	0.0260 (11)	0.0196 (12)	0.0256 (11)	0.0007 (9)	−0.0054 (9)	0.0020 (9)
C18	0.0253 (11)	0.0220 (12)	0.0304 (12)	0.0001 (9)	−0.0032 (9)	0.0081 (9)
C19	0.0217 (11)	0.0237 (12)	0.0316 (12)	0.0019 (9)	−0.0023 (9)	0.0045 (9)
C20	0.0288 (11)	0.0182 (11)	0.0242 (11)	0.0019 (9)	−0.0062 (9)	0.0021 (9)
C21	0.0276 (11)	0.0243 (12)	0.0264 (11)	0.0000 (9)	−0.0023 (9)	0.0056 (9)
C22	0.0221 (11)	0.0256 (12)	0.0322 (12)	0.0030 (9)	−0.0016 (9)	0.0029 (10)
C23	0.0243 (11)	0.0261 (12)	0.0334 (12)	0.0071 (9)	0.0000 (9)	0.0065 (10)
C24	0.0302 (12)	0.0335 (14)	0.0387 (13)	0.0049 (11)	0.0032 (10)	0.0158 (11)
C25	0.0268 (12)	0.0255 (12)	0.0344 (12)	0.0021 (9)	−0.0048 (9)	0.0081 (10)
C26	0.0361 (13)	0.0385 (15)	0.0585 (16)	0.0036 (11)	−0.0067 (12)	0.0212 (12)
C27	0.0306 (12)	0.0448 (15)	0.0375 (13)	0.0069 (11)	−0.0061 (10)	0.0074 (11)

### Geometric parameters (Å, °)

**Table:**

O1—C4	1.355 (2)	C11—H11C	0.9600
O1—C7	1.441 (2)	C12—C13	1.494 (2)
O2—C6	1.374 (2)	C12—H12A	0.9700
O2—C12	1.419 (2)	C12—H12B	0.9700
O3—C13	1.215 (2)	C14—C15	1.469 (3)
O4—C13	1.316 (2)	C15—C16	1.326 (3)
O4—H4	0.8200	C15—H15	0.9300
O5—C14	1.236 (2)	C16—C17	1.464 (3)
O6—C20	1.362 (2)	C16—H16	0.9300
O6—C23	1.442 (2)	C17—C18	1.387 (2)
C1—C2	1.391 (2)	C17—C22	1.396 (3)
C1—C6	1.407 (2)	C18—C19	1.378 (3)
C1—C14	1.494 (3)	C18—H18	0.9300
C2—C3	1.378 (3)	C19—C20	1.389 (3)
C2—H2	0.9300	C19—H19	0.9300
C3—C4	1.381 (2)	C20—C21	1.384 (3)
C3—H3	0.9300	C21—C22	1.380 (3)
C4—C5	1.387 (2)	C21—H21	0.9300
C5—C6	1.385 (2)	C22—H22	0.9300
C5—H5	0.9300	C23—C24	1.496 (3)
C7—C8	1.497 (3)	C23—H23A	0.9700
C7—H7A	0.9700	C23—H23B	0.9700
C7—H7B	0.9700	C24—C25	1.318 (3)
C8—C9	1.320 (3)	C24—H24	0.9300
C8—H8	0.9300	C25—C27	1.491 (3)
C9—C10	1.497 (3)	C25—C26	1.504 (3)
C9—C11	1.501 (3)	C26—H26A	0.9600
C10—H10A	0.9600	C26—H26B	0.9600
C10—H10B	0.9600	C26—H26C	0.9600
C10—H10C	0.9600	C27—H27A	0.9600
C11—H11A	0.9600	C27—H27B	0.9600
C11—H11B	0.9600	C27—H27C	0.9600
			
C4—O1—C7	117.34 (14)	O3—C13—C12	124.87 (17)
C6—O2—C12	117.60 (15)	O4—C13—C12	110.40 (16)
C13—O4—H4	109.5	O5—C14—C15	119.55 (17)
C20—O6—C23	117.28 (14)	O5—C14—C1	118.69 (17)
C2—C1—C6	115.70 (17)	C15—C14—C1	121.75 (16)
C2—C1—C14	115.69 (16)	C16—C15—C14	121.55 (17)
C6—C1—C14	128.60 (17)	C16—C15—H15	119.2
C3—C2—C1	124.82 (18)	C14—C15—H15	119.2
C3—C2—H2	117.6	C15—C16—C17	126.66 (18)
C1—C2—H2	117.6	C15—C16—H16	116.7
C2—C3—C4	117.89 (18)	C17—C16—H16	116.7
C2—C3—H3	121.1	C18—C17—C22	117.20 (18)
C4—C3—H3	121.1	C18—C17—C16	121.73 (17)
O1—C4—C3	125.01 (17)	C22—C17—C16	121.05 (17)
O1—C4—C5	115.13 (16)	C19—C18—C17	122.12 (18)
C3—C4—C5	119.87 (18)	C19—C18—H18	118.9
C6—C5—C4	121.10 (17)	C17—C18—H18	118.9
C6—C5—H5	119.4	C18—C19—C20	119.68 (18)
C4—C5—H5	119.4	C18—C19—H19	120.2
O2—C6—C5	121.04 (16)	C20—C19—H19	120.2
O2—C6—C1	118.32 (17)	O6—C20—C21	116.85 (17)
C5—C6—C1	120.63 (17)	O6—C20—C19	123.79 (17)
O1—C7—C8	106.76 (15)	C21—C20—C19	119.37 (18)
O1—C7—H7A	110.4	C22—C21—C20	120.17 (18)
C8—C7—H7A	110.4	C22—C21—H21	119.9
O1—C7—H7B	110.4	C20—C21—H21	119.9
C8—C7—H7B	110.4	C21—C22—C17	121.45 (18)
H7A—C7—H7B	108.6	C21—C22—H22	119.3
C9—C8—C7	124.88 (19)	C17—C22—H22	119.3
C9—C8—H8	117.6	O6—C23—C24	107.45 (15)
C7—C8—H8	117.6	O6—C23—H23A	110.2
C8—C9—C10	122.93 (19)	C24—C23—H23A	110.2
C8—C9—C11	121.79 (19)	O6—C23—H23B	110.2
C10—C9—C11	115.28 (18)	C24—C23—H23B	110.2
C9—C10—H10A	109.5	H23A—C23—H23B	108.5
C9—C10—H10B	109.5	C25—C24—C23	125.31 (18)
H10A—C10—H10B	109.5	C25—C24—H24	117.3
C9—C10—H10C	109.5	C23—C24—H24	117.3
H10A—C10—H10C	109.5	C24—C25—C27	123.09 (19)
H10B—C10—H10C	109.5	C24—C25—C26	122.65 (19)
C9—C11—H11A	109.5	C27—C25—C26	114.26 (17)
C9—C11—H11B	109.5	C25—C26—H26A	109.5
H11A—C11—H11B	109.5	C25—C26—H26B	109.5
C9—C11—H11C	109.5	H26A—C26—H26B	109.5
H11A—C11—H11C	109.5	C25—C26—H26C	109.5
H11B—C11—H11C	109.5	H26A—C26—H26C	109.5
O2—C12—C13	108.73 (15)	H26B—C26—H26C	109.5
O2—C12—H12A	109.9	C25—C27—H27A	109.5
C13—C12—H12A	109.9	C25—C27—H27B	109.5
O2—C12—H12B	109.9	H27A—C27—H27B	109.5
C13—C12—H12B	109.9	C25—C27—H27C	109.5
H12A—C12—H12B	108.3	H27A—C27—H27C	109.5
O3—C13—O4	124.73 (17)	H27B—C27—H27C	109.5
			
C6—C1—C2—C3	−0.1 (3)	C6—C1—C14—O5	−173.38 (18)
C14—C1—C2—C3	−178.72 (18)	C2—C1—C14—C15	−173.64 (17)
C1—C2—C3—C4	0.0 (3)	C6—C1—C14—C15	7.9 (3)
C7—O1—C4—C3	−1.5 (3)	O5—C14—C15—C16	4.4 (3)
C7—O1—C4—C5	178.51 (17)	C1—C14—C15—C16	−176.88 (19)
C2—C3—C4—O1	179.78 (18)	C14—C15—C16—C17	177.71 (18)
C2—C3—C4—C5	−0.2 (3)	C15—C16—C17—C18	−0.9 (3)
O1—C4—C5—C6	−179.55 (17)	C15—C16—C17—C22	−179.1 (2)
C3—C4—C5—C6	0.5 (3)	C22—C17—C18—C19	1.0 (3)
C12—O2—C6—C5	9.4 (2)	C16—C17—C18—C19	−177.29 (18)
C12—O2—C6—C1	−171.72 (16)	C17—C18—C19—C20	−0.4 (3)
C4—C5—C6—O2	178.39 (17)	C23—O6—C20—C21	176.04 (16)
C4—C5—C6—C1	−0.5 (3)	C23—O6—C20—C19	−3.7 (3)
C2—C1—C6—O2	−178.62 (16)	C18—C19—C20—O6	179.39 (18)
C14—C1—C6—O2	−0.2 (3)	C18—C19—C20—C21	−0.4 (3)
C2—C1—C6—C5	0.3 (3)	O6—C20—C21—C22	−179.27 (17)
C14—C1—C6—C5	178.73 (19)	C19—C20—C21—C22	0.5 (3)
C4—O1—C7—C8	174.14 (16)	C20—C21—C22—C17	0.1 (3)
O1—C7—C8—C9	179.36 (18)	C18—C17—C22—C21	−0.8 (3)
C7—C8—C9—C10	−0.1 (3)	C16—C17—C22—C21	177.45 (18)
C7—C8—C9—C11	−179.8 (2)	C20—O6—C23—C24	178.69 (16)
C6—O2—C12—C13	171.66 (15)	O6—C23—C24—C25	−170.7 (2)
O2—C12—C13—O3	4.4 (3)	C23—C24—C25—C27	−1.1 (3)
O2—C12—C13—O4	−175.26 (14)	C23—C24—C25—C26	178.5 (2)
C2—C1—C14—O5	5.0 (3)		

### Hydrogen-bond geometry (Å, °)

**Table:**

*D*—H···*A*	*D*—H	H···*A*	*D*···*A*	*D*—H···*A*
O4—H4···O3^i^	0.82	1.83	2.6474 (19)	176
C3—H3···O5^ii^	0.93	2.56	3.456 (2)	162

Symmetry codes: (i) −*x*, −*y*+1, −*z*+2; (ii) −*x*+2, −*y*, −*z*+2.
